# Pharmacology and Rationale for Seralutinib in the Treatment of Pulmonary Arterial Hypertension

**DOI:** 10.3390/ijms241612653

**Published:** 2023-08-10

**Authors:** Soni Savai Pullamsetti, Ravikumar Sitapara, Robin Osterhout, Astrid Weiss, Laura L. Carter, Lawrence S. Zisman, Ralph Theo Schermuly

**Affiliations:** 1 Lung Vascular Epigenetics, Center for Infection and Genomics of the Lung (CIGL), Justus-Liebig-Universität Gießen, Aulweg 132, 35392 Giessen, Germany; soni.pullamsetti@innere.med.uni-giessen.de; 2 Gossamer Bio, Inc., San Diego, CA 92121, USA; 3 UGMLC Pulmonale Pharmakotherapie, Biomedizinisches Forschungszentrum Seltersberg (BFS), Justus-Liebig-Universität Gießen, Schubertstraße 81, 35392 Giessen, Germany; astrid.weiss@innere.med.uni-giessen.de; 4 Department of Internal Medicine, Justus-Liebig-University Giessen, Aulweg 130, 35392 Giessen, Germany

**Keywords:** PDGFR, c-KIT, CSF1R, ABL, imatinib, dasatinib, inhalation

## Abstract

Pulmonary arterial hypertension (PAH) is a complex disorder characterized by vascular remodeling and a consequent increase in pulmonary vascular resistance. The histologic hallmarks of PAH include plexiform and neointimal lesions of the pulmonary arterioles, which are composed of dysregulated, apoptosis-resistant endothelial cells and myofibroblasts. Platelet-derived growth factor receptors (PDGFR) α and β, colony stimulating factor 1 receptor (CSF1R), and mast/stem cell growth factor receptor kit (c-KIT) are closely related kinases that have been implicated in PAH progression. In addition, emerging data indicate significant crosstalk between PDGF signaling and the bone morphogenetic protein receptor type 2 (BMPR2)/transforming growth factor β (TGFβ) receptor axis. This review will discuss the importance of the PDGFR-CSF1R-c-KIT signaling network in PAH pathogenesis, present evidence that the inhibition of all three nodes in this kinase network is a potential therapeutic approach for PAH, and highlight the therapeutic potential of seralutinib, currently in development for PAH, which targets these pathways.

## 1. Introduction to Pulmonary Arterial Hypertension

Pulmonary arterial hypertension (PAH) is a complex disorder characterized by vascular remodeling and a consequent increase in pulmonary vascular resistance (PVR). According to the 6th World Symposium on Pulmonary Hypertension, PAH is defined hemodynamically as a mean pulmonary arterial pressure (mPAP) greater than or equal to 20 mm Hg, with a pulmonary capillary wedge pressure less than or equal to 15 mm Hg and PVR greater than 3 Wood units [1].

The histologic hallmarks of PAH include plexiform and neointimal lesions of the pulmonary arterioles, which are composed of dysregulated, apoptosis-resistant endothelial cells and myofibroblasts [2,3]. The pulmonary artery smooth muscle cells (PASMCs) within these lesions undergo hypertrophy and proliferate, resulting in abnormally muscularized pulmonary arterioles. There is also a growing body of evidence highlighting a key role of inflammation in PAH, wherein perivascular inflammatory cells consisting of T cells, B cells, macrophages, and mast cells establish a milieu for abnormal vascular remodeling [3,4]. For instance, perivascular macrophages and mast cells stimulate PASMC proliferation via the aberrant paracrine secretion of growth factors [3,4,5]. Signaling through several tyrosine kinases, including platelet-derived growth factor receptor (PDGFR) α, PDGFRβ, colony stimulating factor 1 receptor (CSF1R), and mast/stem cell growth factor receptor kit (c-KIT), contribute to the cycle of inflammation and proliferation involved in PAH pathogenesis and progression. Therefore, these pathways have gained attention as potential targets for therapeutic intervention. Prior clinical studies with imatinib, an Abelson (ABL), PDGFR, and c-KIT tyrosine kinase inhibitor, indicated that targeting one or more of these pathways could be effective [6]. Although oral imatinib demonstrated efficacy in clinical trials, its further development was limited by systemic side effects.

## 2. Seralutinib: Mode of Action, Pharmacology, and Pharmacokinetics

**Figure 1:**
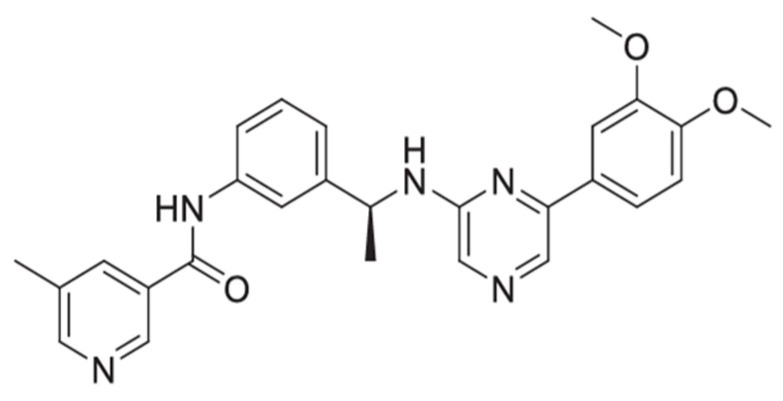
Structure of seralutinib (formerly known as GB002).

Recently, the phase 2 multicenter, double-blind, randomized, placebo-controlled study of inhaled seralutinib in World Health Organization (WHO) Group I pulmonary hypertension (PH) (TORREY, NCT04456998) met its primary endpoint of reducing PVR after 24 weeks of treatment. Seralutinib is a small-molecule, highly potent PDGFRα/PDGFRβ, CSF1R, and c-KIT kinase inhibitor specifically designed for inhalation to treat PAH locally with limited systemic exposure (Figure 1) [7]. Seralutinib is a hydrophobic compound with limited oral bioavailability, features desirable for an inhaled product. It is delivered by an RS01 dry powder inhaler and has aerosol properties designed to deliver the dose to the deep lung, while limiting systemic exposure. These features of seralutinib have been confirmed preclinically in rats, where passively inhaled seralutinib achieved ~30-fold higher lung exposure compared to plasma based on area under the concentration time curves at a pre-clinical efficacious dose [7]. In addition, seralutinib inhalation significantly inhibited PDGF-BB-induced PDGFRα/β autophosphorylation in the rat lung.

In preclinical studies, seralutinib inhibited the PDGF-BB-induced proliferation of H1703 cells (a PDGFRα-driven lung cancer cell line), human PASMCs (which express similar levels of PDGFRα and PDGFRβ), and human lung fibroblasts (HLF, which express higher levels of PDGFRβ than PDGFRα) with half maximal inhibitory concentrations (IC_50_) of 32 nM, 33 nM, and 29 nM, respectively (Table 1) [7]. Although imatinib inhibited H1703 cell proliferation with an IC_50_ of 62 nM, its potency was 13-to-20-fold lower than seralutinib in human PASMC and HLF proliferation assays [7]. Both compounds potently inhibited PDGF-BB-induced extracellular signal-regulated kinase (ERK) phosphorylation in H1703 cells; however, only seralutinib inhibited ERK phosphorylation in HLFs. Furthermore, seralutinib was more potent than imatinib against c-KIT and CSF1R kinases. Seralutinib inhibited stem-cell factor (SCF)-induced c-KIT autophosphorylation in human pulmonary artery endothelial cells (HPAECs) with an IC_50_ of 7.8 nM, and displayed an IC_50_ of 14.4 nM in macrophage colony-stimulating factor (M-CSF)-induced CSF1R autophosphorylation in primary human macrophages (a 34-fold greater potency versus imatinib) [7]. Notably, imatinib inhibits c-abl kinase, while seralutinib does not. C-abl signaling is critical for PAEC apoptosis, and decreased c-abl signaling is associated with pulmonary endothelial dysfunction in PAH [8].

## 3. Rationale for Seralutinib for the Treatment of PAH

### 3.1. Overview of Type III RTKs

**Figure 2:**
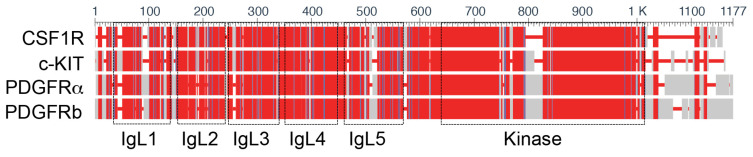
The type III tyrosine kinase receptor family includes platelet-derived growth factor receptor α (PDGFRα) and β (PDGFRβ), tyrosine-protein kinase kit (c-KIT), and colony-stimulating factor 1 receptor (CSF1R). These receptors display high homology and arise from the tandem duplication of an ancestral type III receptor tyrosine kinase. Alignment performed by COBALT (constraint-based alignment tool) (BLAST multiple alignment) [11] and colored by the conservation of amino acid positions (red = conserved; blue = not conserved; grey = alignment gaps). Domain annotations of immunoglobulin-like (IgL) and kinase regions are from Uniprot.

The type III tyrosine kinase receptor (RTK) family includes PDGFRα, PDGFRβ, c-KIT, and CSF1R. These receptors arose as a result of a tandem duplication from an ancestral type III receptor tyrosine kinase [9]. Members of this receptor family contain five extracellular immunoglobulin (Ig)-like domains, a single transmembrane domain, an intracellular kinase domain, and a carboxy tail (Figure 2) [9]. PDGF ligands (encoded by PDGFA, PDGFB, PDGFC, and PDGFD genes) form homo- and heterodimers that display differential affinity towards the different PDGFR complexes (PDGFRα/α, PDGFRα/β, or PDGFRβ/β). PDGFAA, PDGFBB, PDGFAB, and PDGFCC display a stronger affinity towards the PDGFRα/α homodimer. PDGFRα/β heterodimer can be bound by PDGFBB and PDGFAB, while PDGFBB and PDGFDD display a preferred affinity towards the PDGFRβ/β homodimer. Stem cell factor (SCF) is the only ligand that has been described for the c-KIT receptor, while both CSF1 and interleukin (IL)-34 are CSF1R ligands [9,10]. In all Type III RTKs, ligand binding induces receptor dimerization and intracellular tyrosine residue autophosphorylation, inducing conformational changes that facilitate the docking of SH2-domain containing adaptor proteins and initiating downstream signal transduction cascades.

**Figure 3:**
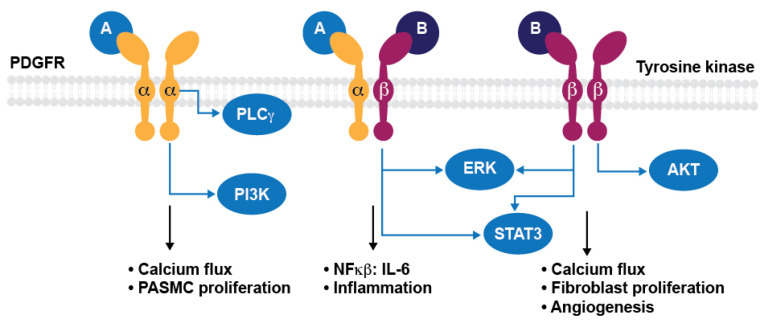
Genetic knockout studies elucidated both overlapping and distinct signal transduction effects of activating PDGFRα/α, α/β or β/β isoforms. In PAH, PDGFRα and PDGFRβ drive pulmonary arterial smooth muscle cell (PASMC) proliferation, while PDGFRβ plays a more prominent role in fibroblast proliferation. AKT, protein kinase B; ERK, extracellular signal-regulated kinase; IL-6, interleukin 6; NFκB, nuclear factor kappa B; PAH, pulmonary arterial hypertension; PASMC, pulmonary artery smooth muscle cells; PDGFR, platelet-derived growth factor receptor; PI3K, phosphoinositide-3-kinase; PLCγ, phospholipase C gamma; STAT3, Signal Transducer and Activator of Transcription 3.

Signaling cascades downstream from PDGF receptors include phosphoinositide-3-kinase (PI3K)/protein kinase B (PI3K/AKT), phospholipase C (PLC)γ, and RAS/MAPK/ERK pathways [12]. PDGFRα/α and PDGFRβ/β homodimer receptor complexes can induce powerful mitogenic signals, while PDGFRβ/β and PDGFRα/β complexes have also been implicated in chemotaxis [13]. Genetic knockout studies have elucidated both overlapping and distinct signal transduction effects of activating PDGFRα/α, α/β or β/β isoforms (Figure 3). For example, PDGFRα/α activated pathways were found to be associated with the inflammatory response (GO:0050728) [12]. PDGFRα/β was found to mediate the activation of nuclear factor kappa B (NFκB) and IL-6 signaling pathways, whereas PDGFRβ/β was found to uniquely activate pathways associated with angiogenesis (GO:0001525) [12]. In addition, PDGF activation of AKT has been shown to enhance store-operated calcium entry and cell proliferation in human PASMCs [14].

### 3.2. The Role of Type III RTKs in PAH Pathogenesis

**Figure 4:**
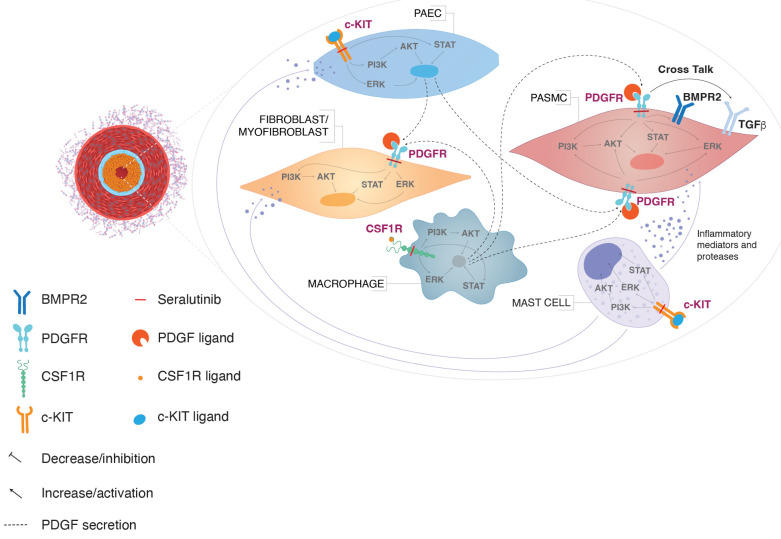
Schematic describing the pathogenic cell circuit and CSF1R, PDGFR, c-KIT and BMPR2 kinase signaling interactions in the PAH microenvironment. Activated CSF1R-positive macrophages and c-KIT-positive pulmonary artery endothelial cells (PAEC) secrete PDGF, which binds to PDGFRs present on the pulmonary artery smooth muscle cells (PASMC) and fibroblasts [7]. The activation of PDGFR induces PI3K, AKT, STAT, and ERK signaling cascade and proliferation of PASMCs and fibroblasts within the pulmonary vascular lesion. This leads to the medial hypertrophy of pulmonary arterioles, and neointimal proliferation and perivascular fibrosis. PDGFR stimulation also leads to the downregulation of BMPR2 and further exacerbates the proliferative process in a reinforcing feedback loop. In addition, the activation of c-KIT in mast cells can further contributes to the inflammatory and abnormal proliferation that leads to pulmonary vascular remodeling. Seralutinib inhibits PDGFR, CSF1R, and c-KIT, and increases BMPR2 levels, thereby modulating key signaling pathways involved in pathological remodeling in pulmonary arterial hypertension. AKT, protein kinase B; BMPR2, bone morphogenetic protein receptor type 2; c-KIT, tyrosine-protein kinase kit; CSF1R, colony-stimulating factor 1 receptor; ERK, extracellular signal-regulated kinase; PAEC, pulmonary artery endothelial cell; PAH, pulmonary arterial hypertension; PASMC, pulmonary artery smooth muscle cell; PDGFR, platelet-derived growth factor receptor; PI3K, phosphoinositide-3-kinase; STAT3, Signal Transducer and Activator of Transcription 3. Distributed under the terms of the Creative Commons Attribution-Non-Commercial 4.0 License; (https://creativecommons.org/licenses/by-nc/4.0/, accessed on 31 January 2023).

Multiple networks of kinase signaling impact the development of neointimal lesions in PAH. These kinases do not operate in isolation, but rather interact in complex networks to exert downstream biological effects. Type III RTKs PDGFRα and PDGFRβ, c-KIT, and CSF1R have each been implicated in the development and/or pathogenesis of PAH, and these RTKs together constitute a pathogenic cell circuit (Figure 4) [7].

**Figure 5:**
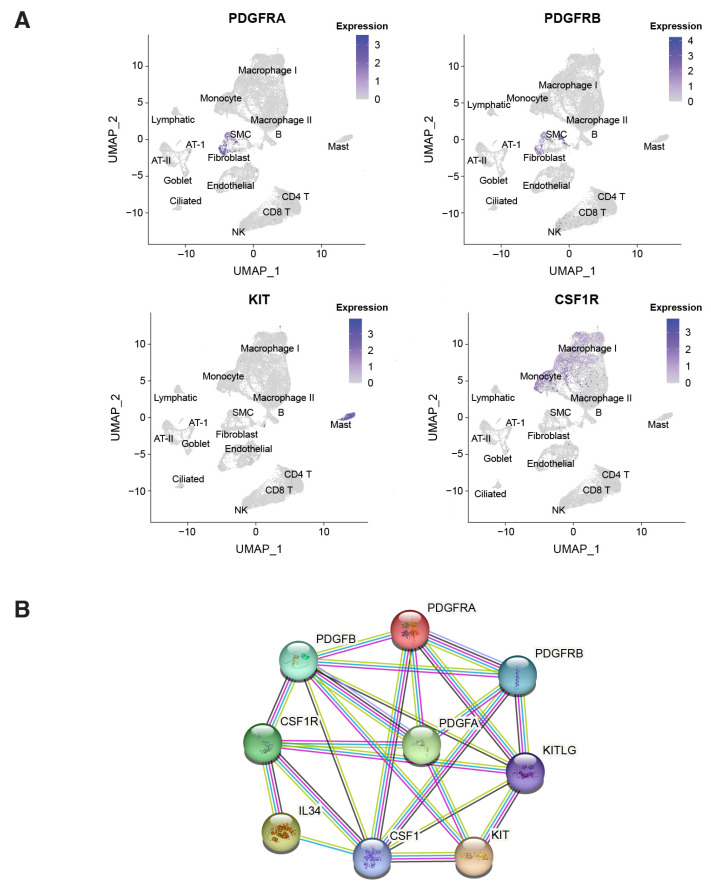
(**A**) Single-cell RNA sequencing data from explanted PAH lungs support cell-type-specific expression of type III RTKs in the lung microenvironment with CSF1R expressed in macrophages, PDGFRα/β expressed in PASMCs and fibroblasts and c-KIT expressed in mast cells. Data were normalized and re-processed following methods described by Saygin, et al. [21]; cluster annotations were confirmed using the Human Lung Cell Atlas reference [22]. (**B**) STRING Network analysis (STRING-db version 11.5) demonstrated close interactions between PDGFR, CSF1R, c-KIT and their associated ligands, with colored lines representing protein–protein interactions at different levels of confidence: pink = experimentally determined, blue = curated databases, green = predicted gene neighborhood, red = predicted gene fusion, blue = predicted gene co-occurrence, yellow = textmining, black = co-expression, purple = protein homology [23]. c-KIT, tyrosine-protein kinase kit; CSF1R, colony stimulating factor 1 receptor; IL34, interleukin 34; PAH, pulmonary arterial hypertension; PASMC, pulmonary artery smooth muscle cell; PDGFR, platelet-derived growth factor receptor; RTK, tyrosine kinase receptor; UMAP, uniform manifold approximation and projection.

PDGF receptors α and β (PDGFRα/β) are predominantly expressed on the surface of PASMCs and myofibroblasts, driving excessive cell proliferation in response to PDGF ligands secreted by endothelial cells and infiltrating macrophages [15]. CSF1R is expressed on monocytes and macrophages, which secrete PDGF ligands and pro-inflammatory cytokines contributing to pathological remodeling in PAH [9,16,17]. Myofibroblasts in turn secrete CSF1, further recruiting and activating CSF1R-positive macrophages in a positive feedback loop. c-KIT is expressed on endothelial progenitor cells and mast cells, potentially contributing to vascular remodeling and perivascular inflammation [18,19,20]. The increased infiltration of c-KIT-positive cells was observed in pulmonary arterial plexiform lesions in PAH patient lungs [19]. The cell-type-specific expression of PDGFRα and PDGFRβ, c-KIT, and CSF1R and their ligands within the PAH lung microenvironment was corroborated by single-cell RNAseq data from human PAH lungs (Figure 5A).

Type III RTKs and their ligands exhibit myriad canonical and non-canonical interactions, supported by experimental evidence and computational predictions using the STRING online web tool; (https://string-db.org, accessed on 31 January 2023) (Figure 5B) [23], further suggesting that crosstalk between signaling components may contribute to PAH pathobiology.

### 3.3. PDGFR Signal Transduction in PAH

A growing body of research has demonstrated a compelling case for targeting PDGF signaling in PAH. The PDGF pathway is markedly upregulated in pulmonary vascular lesions from PAH patients [7,15]. PDGFA, PDGFB, PDGFRα and PDGFRβ mRNA and protein expression were increased in pulmonary arterioles. Western blot analysis showed a significant increase in PDGFRβ protein and quantitative immunohistochemistry demonstrated increased PDGFRα and PDGFRβ in PAH lungs [7,15]. In the pulmonary arterioles, PDGFA and PDGFB were mainly localized to PASMCs and PAECs [15]. In addition, PDGFA and PDGFB showed intense staining in perivascular infiltrates. PDGFRα and PDGFRβ mainly stained PASMCs, and to a lesser extent endothelial cells. Notably, the PDGFB gene was recently found to be the most upregulated cardiovascular gene in idiopathic PAH [21]. In addition, TYKRIL, a long noncoding RNA, was recently found to be upregulated in PAH and shown to induce PDGFRβ expression [24]. Moreover, a recent genetic study of rare variants in PAH patients identified platelet-derived growth factor D (PDGFD) as a new candidate risk gene in adult-onset idiopathic PAH, further implicating PDGFR signaling in PAH pathobiology [25].

Preclinical and clinical studies support the role of PDGFR signaling in PAH disease pathobiology. In vitro, the protein expression of PDGFRα and β was upregulated in idiopathic PAH (IPAH) PASMCs, and the PDGF-mediated activation of PDGFRα and PDGFRβ was enhanced in IPAH PASMCs compared to normal controls [26]. Levels of PDGFAA and PDGFBB ligands were also increased in conditioned media from IPAH PAECs compared to normal PAECs. The incubation of IPAH PASMCs with conditioned media from IPAH PAECs activated PDGFRα in IPAH PASMCs and induced greater proliferation compared to conditioned media from normal PAECs, providing evidence for paracrine crosstalk between the two cell types. In vivo, tyrosine 739, 750, and 1020 point mutations in PDGFRβ that interfere with PDGF-dependent PI3K and PLCγ activity prevented hypoxia-induced pulmonary hypertension in a mouse model [27]. Lineage tracing studies have identified PDGFRβ/smooth muscle cell (SMC) marker-positive progenitors, located at the muscular arteriole border in the normal lung and in hypoxia-induced pulmonary hypertension, that give rise to most of the pathological muscularization of small arterioles [28]. Furthermore, multiple preclinical animal studies have demonstrated that PDGFR inhibition could be effective in models of PAH [7,29,30]. In the clinical setting, imatinib (cABL/PDGFR/c-KIT tyrosine kinase inhibitor) demonstrated efficacy in a phase 3 study with a robust effect on PVR and 6 min walk distance (6MWD), but was limited by its safety and tolerability profile [6].

### 3.4. CSF1R Signaling and Potential Importance in PAH

Macrophages, which express CSF1R, are now recognized to play an important role in PAH pathology [31]. Several lines of evidence support this hypothesis. Activated CSF1R+ macrophages accumulate around pulmonary arterioles in PAH, and quantitative immunohistochemistry (IHC) has shown increased CSF1R+ cells in PAH [7,32]. Positron emission tomography has been used to show that inflammation due to macrophages occurs in vivo in PAH patients [33,34].

Experimental models of PH are associated with the accumulation of CD68+ macrophages, and the depletion of these macrophages can prevent or reverse PH [33,35]. For example, in a murine hypoxia model of PH, “alternative” macrophage activation was essential for vascular remodeling [34,35]. In an animal model of hepatopulmonary syndrome, CD68+ macrophages accumulated in pulmonary arteries in part due to increased monocyte chemoattractant protein-1 (MCP-1), chemokine (C-C motif) and ligand 2 (CCL2), and macrophage depletion reversed the hemodynamic and histologic features of the disease [36].

CSF1R inhibition has been shown to decrease the adhesion of macrophages. It has been proposed that the mechanism by which CSF1R inhibition decreases macrophage adhesion is through its effects on PI3K and subsequent paxillin activation [37]. CSF1R inhibition does not appear to directly affect M1 or M2a macrophage polarization, but was found to increase chemokine (C-C motif) ligand 18 (CCL18) gene expression in M2a macrophages, and to increase CCL22 as well as PDGFB gene expression without affecting the secretion of their respective proteins [37]. In addition, CSF1R inhibition decreased CSF1-stimulated CCL2 gene expression. It has been proposed that this decrease in CCL2 mediated by CSF1R inhibition could be, for example, a mechanism to treat pulmonary fibrosis [37]. The CCL2 receptor, CCR2, may mediate crosstalk between macrophages and pulmonary artery smooth muscle cells in PAH [16]. Therefore, CSF1R inhibition could also alter interactions of macrophages and PASMCs through its effects on CCL2.

The CSF1 receptor is activated by the homo dimeric growth factors CSF1 and IL-34. CSF1 can be detected in the circulation, but IL-34 is not, and thus its actions are likely restricted to the local microenvironments in which it is expressed. The PI3K/AKT pathway plays a central role in CSF1-mediated macrophage survival. Macrophage proliferation is associated with a CSF1 dose-dependent increase in protein synthesis. The CSF1R phosphorylation of tyrosine residue 807 activates both the MEK and PI3K pathways that independently lead to macrophage proliferation. In addition, CSF1 stimulates a dynamic reorganization of the actin cytoskeleton, which can enhance macrophage migration [9].

Computational and experimental approaches have been used to characterize a cell circuit between macrophages and fibroblasts. In one model, cell–cell contact was essential for the population stability of the macrophage–fibroblast circuit [38]. This circuit was kept stable by paracrine signaling between the two cell types. Fibroblasts were identified to be a source of CSF1 ligand; however, only macrophages expressed CSF1R. Similarly, while macrophages secreted PDGF, only fibroblasts were responsive to the PDGF ligand via the expression of PDGF receptors [38]. The importance of this crosstalk has been confirmed in several preclinical models of lung fibrosis, where CSF1R+ macrophages have been implicated in orchestrating the development of a lung fibrotic niche [17,39,40]. The recruitment of monocyte-derived macrophages to sites of lung tissue damage has been shown in models of radiation-, asbestos-, and bleomycin-induced fibrosis [17,40]. Single-cell transcriptomic analysis of lungs from mice treated with asbestos or bleomycin and patients with pulmonary fibrosis revealed CSF1/CSF1R signaling as one of the key factors controlling monocyte-derived alveolar macrophages. The pharmacological blockade of pro-fibrotic macrophages with either anti-CSF1R Ab, CSF1 Ab or the small molecule CSF1R inhibitor, PLX3397, decreased the number of monocyte-derived alveolar macrophages and decreased the severity of pulmonary fibrosis, providing evidence for modulation of the macrophage–fibroblast circuit via CSF1R inhibition in vivo. These data suggest that the macrophage–fibroblast circuit is a key node in fibrotic lung disease, and interference with this crosstalk may have a significant impact on pathological disease remodeling. A similar circuit has been proposed for macrophages and PASMCs in PAH [16]. As previously mentioned, the exposure of PASMCs to conditioned media from in vitro differentiated macrophages resulted in increased PASMC growth, especially with conditioned media from M2 differentiated conditions. Exposure of PASMCs to conditioned media from macrophage–PASMC co-cultures potentiated the growth response even further. These studies identified the CCL2/CCR2 and CCL5/CCR5 axis as a key pathway in macrophage and PASMC crosstalk in vitro and in vivo, demonstrating that recruitment of macrophages can have an impact on PAH progression in preclinical models [16].

Sheikh et al. [41] characterized macrophage–PASMC crosstalk and the involvement of the CSF1R-PDGFR circuit in PAH pathophysiology. Although both endothelial cells and macrophages secrete PDGF-BB, PDGFB deletion in CSF1R positive monocyte/macrophage cells substantially impacted distal pulmonary arteriole muscularization in the hypoxia-induced pulmonary hypertension mouse model, suggesting that the PDGF secreted by macrophages was integral to vascular remodeling. Hypoxia-inducible factor (HIF)1α and HIF2α were found to be directly upstream of PDGFB in macrophages, as the deletion of either gene in macrophages under hypoxic conditions displayed similar effects as PDGFB deletion [42]. Gain-of-function studies further corroborated the regulation of PDGFB expression by HIF1α. Furthermore, conditioned media from macrophages isolated from IPAH or systemic sclerosis (SSc)-PAH patients induced human PASMC proliferation and migration in a PDGF-BB dependent manner, providing evidence for the importance of this circuit in human PAH [42]. Interestingly, fate-mapping studies showed that approximately 10% of hypoxia-induced PDGFRβ-positive distal PASMCs also expressed CD68, suggesting that recruited monocytes can transdifferentiate into smooth muscle cells and directly contribute to distal arteriolar muscularization [41].

Macrophage activity in PAH is associated with bone morphogenetic receptor type 2 (BMPR2) levels [43]. A decrease in BMPR2 characteristic of PAH results in the induction of granulocyte-macrophage colony stimulating factor and macrophage recruitment [44]. Notably, studies in a BMPR2 knockout mouse model showed significant pulmonary inflammation due to the activation of tissue macrophages. When activated with lipopolysaccharide, both mutant and wild-type macrophages secreted BMP pathway inhibitors and are suggested to be sufficient to suppress BMP pathway activity in smooth muscle cells. The co-culturing of smooth muscle cells with macrophages resulted in a BMP signaling-dependent increase in scratch closure [43]. Aside from directly impacting smooth muscle cell proliferation, macrophages can also contribute to extracellular matrix remodeling in PAH via the secretion of legumain, influencing matrix metalloproteinase activation, and altering transforming growth factor β1 (TGFβ1) processing [43,45]. Thus, macrophages play a key role in the inflammation, hyperproliferation and fibrosis that characterize PAH, and the modulation of their cellular functions could be useful in targeting PAH disease remodeling.

### 3.5. c-KIT Signaling and Potential Importance in PAH

c-KIT, the transmembrane receptor kinase for the progenitor SCF, has been implicated in the dysregulated function of mast cells and endothelial cells in vascular disease and PAH [18,19,20]. c-KIT expression has also been associated with vascular remodeling in PAH [19]. c-KIT is a marker of hematopoietic progenitor cells, which are dependent on SCF/c-KIT signaling for proliferation and survival. c-KIT expression is typically lost upon cell differentiation and maturation, with the exception of mast cells and dendritic cells, which express elevated c-KIT even as fully differentiated cells [46]. SCF dose-dependently promoted survival, migration, and capillary tube formation of human umbilical vein endothelial cells [47]. Furthermore, c-KIT expression and SCF activity were increased in endothelial progenitor cells (EPC), translating to substantially enhanced EPC-mediated neovascularization in the presence of SCF in vivo, as compared to mature endothelial cells [48].

Vascular injury studies conducted by Wang et al. evaluated the role of c-KIT in vascular smooth muscle cells (VSMCs) and demonstrated increased expression of c-KIT upon vascular injury in vitro and in vivo [49]. The administration of SCF in vitro lowered the amounts of cleaved caspase and reduced smooth muscle cell apoptosis. In vivo, SCF deficiency disrupted the ability of the SCF/c-KIT system to rescue injured VSMCs, as indicated by extensive apoptotic events throughout all three layers of the injured vessel wall. Similarly, more extensive apoptosis was observed in the injured vessel wall in c-KIT mutant mice, which significantly reduced intimal hyperplasia.

Hypoxia has also been shown to increase c-KIT expression on endothelial cells, enhancing the angiogenic response of endothelial cells to SCF and contributing to pathological vascular remodeling in several different models, including the SU5416 Hypoxia rat PAH model [50,51].

**Figure 6:**
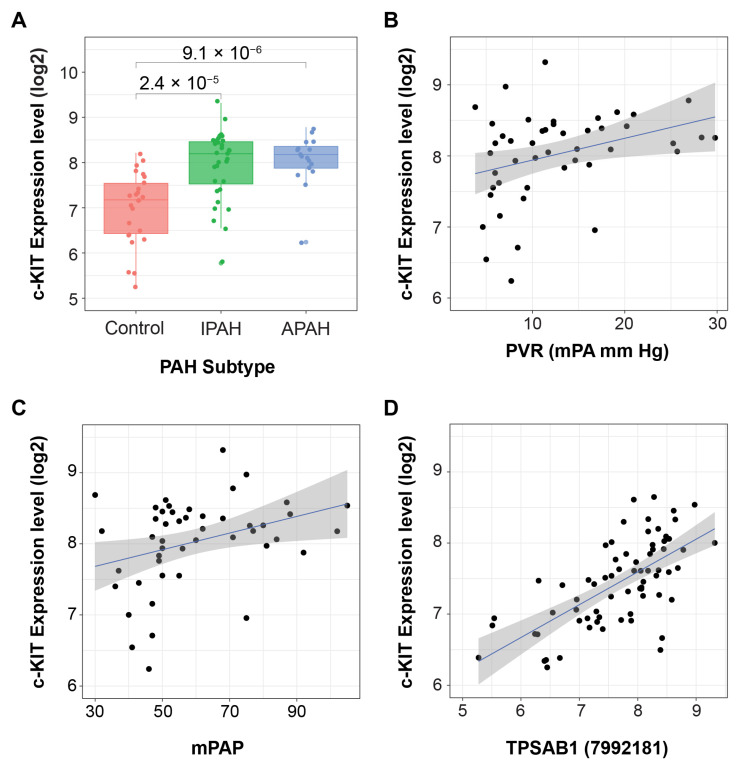
In human lung transcriptome profiles from control, IPAH and APAH patients, c-KIT mRNA was upregulated in PAH lung tissue relative to control lungs, and was significantly correlated with pulmonary vascular resistance (PVR), mean pulmonary artery pressure (mPAP), and tryptase alpha/beta 1 (TPSAB1) mRNA. (**A**) Expression level of c-KIT in idiopathic PAH (IPAH), associated PAH (APAH), and control lungs. (**B**–**D**) c-KIT is positively correlated with PVR (R = 0.34, *p* = 0.021), mPAP (R = 0.34, *p* = 0.001) and the expression of mast cell marker TPSAB1 (probe ID 7998434) (R = 0.66, *p* = 4.4 × 10^−10^). Expression data from Stearman et al. [52] were acquired from the Gene Expression Omnibus (accession GSE117261). *p*-values between groups were determined by unpaired *t*-test. Correlation statistics were calculated using the Pearson method and shaded grey areas around regression lines represent standard error.

Increased infiltration of c-KIT positive cells was observed in pulmonary arterial plexiform lesions in PAH patient lungs across different studies [7,18,19,20]. Immunohistochemistry in PAH lung samples has shown that both c-KIT-positive/tryptase-positive mast cells and c-KIT-positive/tryptase-negative bone marrow-derived cells are increased in pulmonary arteries of patients with IPAH compared with control subjects [19]. The analysis of transcriptome profiles from human lung tissues from associated PAH (APAH) and IPAH patients [52] confirmed the significantly elevated expression of c-KIT in diseased lung tissues compared to controls (failed donors) (Figure 6A), which correlated with mPAP and PVR (Figure 6B,C). The expression of c-KIT displayed significant correlation with the expression of the mast cell marker tryptase α,β1 (TPSAB1, Figure 6D), suggesting that the signal was coming predominantly from mast cells. This finding was corroborated in a human PAH lung scRNAseq dataset showing that c-KIT is predominantly expressed in the mast cell subset (Figure 5A) [21].

c-KIT-positive mast cells secrete pro-inflammatory cytokines and tryptase that further contribute to the inflammatory process in PAH. The excessive influx and degranulation of mast cells in lung tissue was reported in the monocrotaline model of PAH [53]. Imatinib, which inhibits PDGFR and c-KIT, was effective in a monocrotaline rat model of PAH, which suggests that targeting PDGFR and c-KIT signaling cascades could have an impact on PAH disease remodeling. In a PAH clinical trial, imatinib decreased tryptase, which is secreted by mast cells [54]. The decrease in tryptase was found to correlate with disease severity. These data suggest that c-KIT inhibition may be contributing to imatinib’s mechanism of action, and that the modulation of tryptase may serve as a potential biomarker of response. c-KIT signaling may be contributing to pathological disease remodeling in PAH, and the localized lung delivery of a c-KIT inhibitor is a logical therapeutic strategy [9].

### 3.6. Interactions between PDGFR and BMPR2/TGFβ

The serine/threonine kinase BMPR2 plays a critical role in the pathogenesis of PAH, and BMPR2 deficiency is associated with a genetic predisposition to the development of PAH [33,44]. The reduced expression of BMPR2 results in exaggerated signaling responses to TGFβ, increasing susceptibility of endothelial cells to apoptosis, and abnormal growth responses of PASMCs to TGFβ and BMP ligands [55]. Even in the absence of BMPR2 mutations, the downmodulation of BMPR2 protein has been reported in PAH [56]. One of the mechanisms of BMPR2 suppression results from PDGFR signaling; crosstalk between these two pathways could play a role in PAH disease remodeling [56,57].

Direct BMPR2 and PDGFR crosstalk in PASMCs was identified by Chen et al. in their study of PASMC response to PDGFBB stimulation using RNA sequencing and proteomics [57]. PDGFBB treatment decreased BMPR2 expression by upregulating miR-376b, a BMPR2-targeting microRNA, which increased the proliferation of PASMCs. This study provided mechanistic evidence that the activation of PDGFR signaling could influence the expression of BMPR2, and demonstrated interconnectivity between two key PAH signaling cascades.

**Figure 7:**
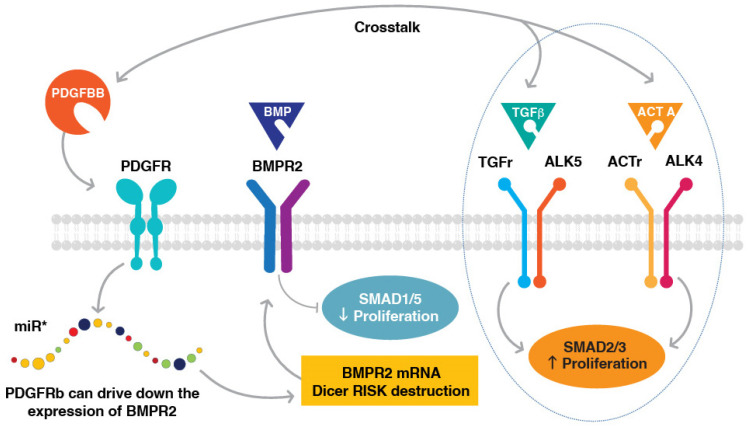
Direct BMPR2 and PDGFR crosstalk in PASMCs. PDGFBB treatment decreased BMPR2 expression by upregulating microRNAs that target BMPR2, thereby increasing the proliferation of PASMCs. * BMPR2-targeting miRNAs include miR-376b, miR-135-5p, and miR-146a-5p [7]. PDGF ligands can also activate signaling through the TGFβ/ALK5 receptor and ACTR/ALK4 receptor complexes resulting in a pro-proliferative response. ACTr, activin receptor type II; ALK, activin receptor-like kinase; BMPR2, bone morphogenetic protein receptor type 2; PASMC, pulmonary artery smooth muscle cell; PDGFR, platelet-derived growth factor receptor; TGFβ, transforming growth factor β; TGFr, TGF beta receptor type I.

The importance of PDGFR and TGFβ signaling crosstalk in PASMCs was also demonstrated by Kudryashova et al., who studied the impact of TGFβ1 and activin A signaling inhibition on PASMC hyperproliferation [58]. PAH PASMCs cells showed the elevated secretion of TGFβ1 and, to a lesser extent, activin A. Inhibitory anti-activin A and anti-TGFβ antibodies reduced the growth of PAH PASMC cells. Although an anti-activin A antibody did not appear to impact canonical (SMAD) or non-canonical (Akt, ERK1/2, p38 MAPK) downstream effectors, an anti-TGFβ antibody significantly reduced the phosphorylation of both SMAD3 and ERK1/2 proteins. Surprisingly, PDGFBB treatment decreased the inhibitory effects of these antibodies. These data demonstrate that PDGFBB signaling could counteract the anti-proliferative effects of therapeutic agents targeting TGFβ1/activin A signaling, and that targeting both PDGF and TGFβ1/activin pathways may be needed to suppress PASMC-driven remodeling processes in PAH (Figure 7).

In the setting of shear stress, the proliferation and migration of endothelial cells and VSMCs were increased due to an increased production of PDGFBB and TGFβ1 [59]. It was found that PDGFBB was involved in the paracrine control of VSMCs by endothelial cells, whereas TGFβ1 played a role in feedback control from VSMCs to endothelial cells [59].

Additional data from other cell types provide further evidence for PDGF and TGFβ receptor crosstalk. In dermal fibroblasts, PDGFBB induced the phosphorylation of SMAD2, which was dependent on the kinase activity of both the TGFβR1 and PDGFRβ [60]. PDGFBB and TGFβ receptors interacted physically in fibroblasts, and stimulation with PDGFBB induced the internalization of both PDGFRβ and TGFβR1. In addition, the silencing of PDGFRβ decreased the stability of TGFβR1 and delayed TGFβR1 signaling [60]. In hepatic stellate cells, PDGFRα was required for TGFβ signaling [61]. The knockdown of PDGFRα inhibited the TGFβ-induced phosphorylation of SMAD2 without an effect on AKT or ERK phosphorylation, and suppressed TGFβR1 gene expression. At the protein level, PDGFRα was recruited to the TGFβR1/2 complex by TGFβ stimulation. Furthermore, PDGFRα knockdown blocked the TGFβ-mediated internalization of TGFβR2, thereby suppressing SMAD2 phosphorylation [61]. Taken together, these data indicate an interdependence of PDGF and TGFβ pathways, and more importantly suggest that the therapeutic targeting of PDGFR may modulate TGFβ signaling in cell types where their receptors may be co-expressed.

### 3.7. Inhibiting a Type III RTK Kinase Network in PAH

**Figure 8:**
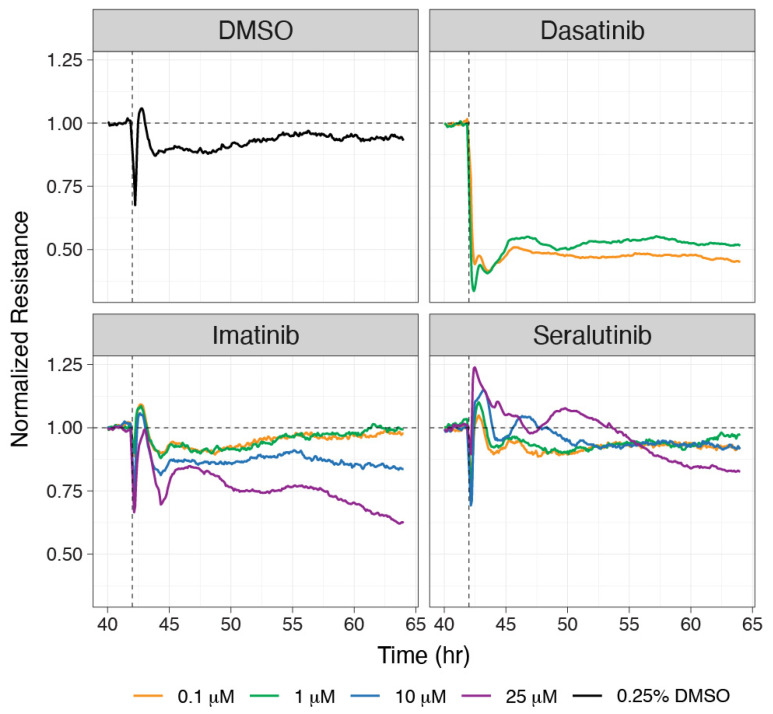
A transendothelial assay was used to examine the effects of 0.25% dimethyl sulfoxide (DMSO), dasatinib, imatinib, and seralutinib on electrical resistance across a monolayer of human pulmonary arterial endothelial cells. Treatment with the drug was started at 42 h after establishing a stable monolayer. Dasatinib showed a dramatic and profound adverse effect on endothelial barrier function, as shown by a severe loss of electrical resistance across the endothelial cell monolayer, whereas seralutinib showed no adverse effects on transendothelial resistance up to 25 µM. Imatinib showed an adverse effect at the high dose of 25 µM.

The role of signaling through PDGFR, CSF1R, and c-KIT in the development and progression of pulmonary vascular remodeling in PAH suggests an opportunity for targeting these pathways as a therapeutic strategy. There has been prior interest in developing tyrosine kinase inhibitors (TKIs) for PAH. However, small-molecule TKIs usually inhibit many different kinases. The kinase specificity of these inhibitors may be helpful in some cases, but the lack of kinase specificity could be harmful. Imatinib is an example of a TKI that held promise as a treatment for PAH. The kinase inhibition profile of imatinib includes PDGFR and c-KIT as well as cABL. In the monocrotaline rat model of PAH, imatinib reversed severe pulmonary hypertension [30]. In a phase 3 trial, imatinib met its primary endpoint of improvement in 6MWD, and significantly improved PVR, but the side effects of orally administrated imatinib presented a limitation for further development [6]. However, given the clinical proof of concept demonstrated for imatinib, new strategies, such as inhalation delivery, are currently in development that have the potential to limit the systemic toxicities observed with oral administration [62]. Nintedanib, a multi-tyrosine kinase inhibitor approved for the treatment of idiopathic pulmonary fibrosis, which targets fibroblast growth factor receptor (FGFR), PDGFR, KIT, and SRC kinases among many others, was also studied in PAH, but failed to improve pulmonary hemodynamics and right heart function in an established animal model and in four patients with severe PAH, and caused the deterioration of these variables in the clinical setting [63]. The use of another broad-spectrum TKI, dasatinib, as a treatment for leukemia has been reported to be associated with pulmonary hypertension and pleural effusion [64,65,66]. It has been suggested that the toxicity associated with dasatinib may be due to an adverse effect on endothelial cell barrier function. In an endothelial cell permeability assay, seralutinib (doses ≤ 25 µM) showed no adverse effects, whereas dasatinib significantly disrupted normal endothelial barrier integrity at 0.1 µM (Figure 8). Imatinib showed a similar adverse effect at a dose of 25 µM. These data suggest that seralutinib could potently inhibit abnormal cell proliferation that is pathognomonic for PAH without altering normal endothelial cell barrier function.

Seralutinib is the first TKI specifically developed as a treatment for PAH, and the first TKI specifically developed for inhalation delivery as a treatment for PAH. Seralutinib was designed to improve potency against PDGFRβ compared to imatinib, inhibit CSF1R and c-KIT, and to have limited oral bioavailability. In contrast to imatinib, seralutinib has limited activity against cABL, but is several orders of magnitude more potent when used for inhibiting CSF1R [7]. While cABL inhibition was a primary goal of imatinib’s use to treat chronic myeloid leukemia (CML), it may represent a liability in the context of treating PAH due to reported cardiotoxicity [67,68]. cABL and phosphorylated cABL levels are lower in the pulmonary vessel endothelium of PAH patients versus controls. Furthermore, in control endothelial cells, the downregulation of cABL by RNA interference resulted in DNA damage and apoptosis, while the restoration of cABL in PAH-derived endothelial cells resulted in reduced DNA damage and apoptosis [8]. On the other hand, it has been proposed that ABL kinase inhibition could protect against vascular leak in certain disease states associated with abnormal endothelial barrier function [69].

Seralutinib’s high potency against CSF1R is a unique feature of this TKI and represents a new approach in targeting this component of the PDGFR/c-KIT/CSF1R kinase network. Preclinical studies in animal models of PAH demonstrated the differential expression of PDGFRα and PDGFRβ signaling in different cell types, and that the potent inhibition of both receptors may be important in targeting this pathway for PAH [29]. Thus, seralutinib’s greater potency against PDGFRβ, for example, could provide a potential advantage compared to imatinib. In addition, seralutinib’s formulation is designed to optimize deep lung delivery via inhalation (using a Plastiape RS01 device), while maintaining rapid clearance to minimize systemic exposure and associated adverse events. The potential therapeutic window for imatinib may be narrower.

Seralutinib delivered by inhalation has shown efficacy in two rat models of PAH: the monocrotaline pneumonectomy (MCT/PN) model and the SU5416 hypoxia (SU5416/H) model [7]. In the MCT/PN model, a telemetry study showed that seralutinib prevented the progression of established PAH, and significantly decreased the extent of neointimal lesions. In the SU5416/H model, a telemetry study showed that seralutinib reversed established PAH, decreased right ventricle hypertrophy, and significantly reversed pulmonary arteriolar remodeling. In a head-to-head comparison with gavage-administered imatinib, inhaled seralutinib showed a greater effect on reducing mPAP and improving pulmonary vascular remodeling in the rat SU5416/H model. In addition, seralutinib decreased N-terminal pro-brain natriuretic peptide (NT-proBNP) and restored BMPR2 levels to normal—effects not observed with imatinib. In a related model in which PDGFB was overexpressed in the lungs followed by further disease induction with SU5416/H, seralutinib showed additive beneficial effects when combined with a phosphodiesterase 5 (PDE-V) inhibitor and an endothelin receptor antagonist [70].

An improvement in anti-inflammatory lung cytokine profiling was also observed in the seralutinib-treated group; there was a 2-fold reduction in circulating tumor necrosis factor α (TNFα) level, and a 4-fold increase in IL-10 level compared to vehicle [7]. These data suggest that seralutinib suppressed pro-inflammatory signaling (e.g., TNFα) and led to the increased expression of protective cytokines (e.g., IL-10).

## 4. Summary and Future Directions

Several novel targeted pathways are under investigation to advance treatment in PAH [71,72,73,74]. Inhibiting PDGFR, CSF1R, and KIT kinases could potentially provide a new therapeutic strategy for the treatment of PAH. These kinases are highly expressed and increased in relevant cell types that characterize pulmonary arteriolar lesions seen in PAH. They interact through autocrine and paracrine signaling to generate inflammation and abnormal cellular proliferation within these lesions. Interest in the potential use of TKIs for PAH was generated by preclinical and clinical proof-of-concept efficacy data with orally administered imatinib; however, systemic side effects were a limitation of this drug via this route of administration. Seralutinib, a novel PDGFR/CSF1R/c-KIT inhibitor administered by inhalation, has unique features compared to imatinib. In particular, seralutinib is more potent against PDGFRβ, and several orders of magnitude more potent against CSF1R compared to imatinib. Furthermore, seralutinib was specifically designed to have low oral bioavailability, a key feature for a drug designed for inhalation. Thus, although it is interesting to consider an inhaled form of imatinib as a treatment for PAH, the oral bioavailability of imatinib and relatively low systemic clearance could limit the therapeutic window of this approach. In contrast, the distinguishing features of seralutinib have the potential to improve both efficacy and tolerability. Seralutinib has shown preclinical efficacy in multiple animal models of PH. Highlighting the crosstalk between PDGFR and BMPR2 signaling, seralutinib restored BMPR2 levels to normal in the SU5416/H model of PH, an effect not observed with imatinib. Future work using techniques such as single cell RNA sequencing could provide further mechanistic understanding of seralutinib’s impact on the kinase network of PDGFR, CSF1R, and c-KIT, and the crosstalk between the different cell subsets involved in pathological remodeling associated with PAH. By targeting PDGFR, CSF1R, and c-KIT, seralutinib could lead to the reverse remodeling of pulmonary vascular disease in PAH, with a consequent improvement in clinically meaningful outcomes with a limited risk for adverse events. Based on its novel mechanism of action and preclinical studies [70], seralutinib may potentially lead to complementary effects when administered with other PAH treatments, but further studies are needed to confirm the benefits of future combination strategies.

In a recent randomized, double-blind, placebo-controlled trial, inhaled seralutinib demonstrated clinical activity, showing a significant reduction in PVR, and was relatively well tolerated after 24 weeks of treatment [75]. Based on this strong scientific rationale and results of the phase 2 TORREY study, a phase 3 study (NCT05934526) will soon begin enrollment to continue the development of seralutinib in PAH.

## Figures and Tables

**Table 1:** In vitro potency of seralutinib versus imatinib [7].

Compound	Biochemical IC_50_ (nM)				Cell-Based IC_50_ (nM) VEK							
	PDGFRα	PDGFRβ *	CSF1R	c-KIT ^†^	H1703 (PDGFRα) ^‡^	HLF (PDGFR β > α) ^§^	HPASMC (PDGFR α = β) ^ll^	H1703 pERK **	HLF pERK **	CSF1R pCSF1R ^††^	CSF1R ^†††^ Reporter Assay	HPAECs pc-KIT ^‡‡^
Seralutinib	7	14	92	20	32	29	33	70	60	14.4	8	7.8
Imatinib	8	75	1160	180	62	579	419	260	>10,000	500	1032	301

* Carna biochemical assay; ^†^ c-KIT biochemical assay; ^‡^ H1703 proliferation assay; ^§^ PDGF-BB-induced HLF proliferation assay; ^ll^ PDGF-BB-induced HPASMC proliferation assay; ** PDGF-BB–induced pERK signaling assay in H1703 and HLF cells; ^††^ M-CSF–induced pCSF1R in primary human macrophages; ^†††^ M-CSF-induced activation of CSF1R-SRE reporter in HEK293; ^‡‡^ SCF-induced phosphorylation of wild type KIT in HPAECs. Distributed under the terms of the Creative Commons Attribution-Non-Commercial 4.0 License; (https://creativecommons.org/licenses/by-nc/4.0/, accessed on 31 January 2023). c-KIT, tyrosine-protein kinase kit; CSF1R, colony stimulating factor 1 receptor; HLF, human lung fibroblast; HPAEC, human pulmonary artery endothelial cell; HPASMC, human pulmonary artery smooth muscle cell; IC_50_, half maximal inhibitory concentration; PDGFR, platelet-derived growth factor receptor; SCF, stem cell factor; M-CSF1, macrophage colony stimulating factor.

## Data Availability

The data presented in this study are openly available.
